# Interactions between *L. monocytogenes* and *P. fluorescens* in Dual-Species Biofilms under Simulated Dairy Processing Conditions

**DOI:** 10.3390/foods10010176

**Published:** 2021-01-16

**Authors:** Francesca Maggio, Chiara Rossi, Clemencia Chaves-López, Annalisa Serio, Luca Valbonetti, Francesco Pomilio, Alessio Pio Chiavaroli, Antonello Paparella

**Affiliations:** 1 Faculty of Bioscience and Technology for Food, Agriculture and Environment, University of Teramo, Via R. Balzarini 1, 64100 Teramo, Italy; fmaggio@unite.it (F.M.); crossi@unite.it (C.R.); cchaveslopez@unite.it (C.C.-L.); aserio@unite.it (A.S.); lvalbonetti@unite.it (L.V.); alessio.chiavaroli27@gmail.com (A.P.C.); 2 Food Hygiene Unit, NRL for *L. monocytogenes*, Istituto Zooprofilattico Sperimentale dell’Abruzzo e del Molise “G. Caporale”, 64100 Teramo, Italy; f.pomilio@izs.it

**Keywords:** *Listeria monocytogenes*, *Pseudomonas fluorescens*, multi-species, biofilms, blue pigment, dairy product

## Abstract

In dairy processing environments, many bacterial species adhere and form biofilms on surfaces and equipment, leading to foodborne illness and food spoilage. Among them, *Listeria monocytogenes* and *Pseudomonas* spp. could be present in mixed-species biofilms. This study aimed to evaluate the interactions between *L. monocytogenes* and *P. fluorescens* in biofilms simulating dairy processing conditions, as well as the capability of *P. fluorescens* in co-culture to produce the blue pigment in a Ricotta-based model system. The biofilm-forming capability of single- and mixed-cultures was evaluated on polystyrene (PS) and stainless steel (SS) surfaces at 12 °C for 168 h. The biofilm biomass was measured, the planktonic and sessile cells and the carbohydrates in biofilms were quantified. The biofilms were also observed through Confocal Laser Scanning Microscopy analysis. Results showed that only *P. fluorescens* was able to form biofilms on PS. Moreover, in dual-species biofilms at the end of the incubation time (168 h at 12 °C), a lower biomass compared to *P. fluorescens* mono-species was observed on PS. On SS, the biofilm cell population of *L. monocytogenes* was higher in the dual-species than in mono-species, particularly after 48 h. Carbohydrates quantity in the dual-species system was higher than in mono-species and was revealed also at 168 h. The production of blue pigment by *P. fluorescens* was revealed both in single- and co-culture after 72 h of incubation (12 °C). This work highlights the interactions between the two species, under the experimental conditions studied in the present research, which can influence biofilm formation (biomass and sessile cells) but not the capability of *P. fluorescens* to produce blue pigment.

## 1. Introduction

Microbial biofilms are three-dimensional structures of various bacteria that adhere to biotic or abiotic surfaces and differentiate into complex communities embedded within extracellular polymeric substances (EPSs) [1]. The relevance of microbial biofilms has been described in different fields including the food industry, where biofilms are responsible for potential food contamination, corrosion, and economic losses [2]. Particularly in the dairy industry, many bacterial species adhere and form biofilms on surfaces and equipment and, among them, *Listeria monocytogenes* and different species of *Pseudomonas* [3] are worthy of attention. 

*Listeria monocytogenes* is a ubiquitous pathogen, able to colonize and persist on common surfaces in the food processing environments, thanks to its biofilm formation capability [4]. This psychrotrophic bacterial pathogen can contaminate a wide variety of foods. In particular, several dairy products such as Blue mold cheese, Camembert cheese, and Ricotta have been implicated in listeriosis outbreaks [5]. An increasing trend for human listeriosis cases has been observed from 2009 to 2018 with 2549 cases reported in Europe in 2018 [6]. Moreover, listeriosis has an important socio-economic impact, which can reach approximately €6327 per case [7].

The microbiota of refrigerated foods is dominated by selected microorganisms, such as *Pseudomonas* spp. In particular, *P. fluorescens* has been isolated from numerous food products including dairy products, as it can colonize and form biofilms onto surfaces of dairy processing plants [2]. Due to the production of hydrolytic enzymes and pigments such as pyoverdine, pyocianin, and indigoidine [8], *P. fluorescens* is responsible for food quality decay, food spoilage, reduced shelf-life [9,10], and defects, as in the case of dairy product blue discoloration [11]. In fact, discoloration due to the microbial activity is not an unusual phenomenon and affects several varieties of cheeses and dairy products made from raw or pasteurized milk [12]. Specifically, *P. fluorescens* produces several pigments, including pyoverdine and indigoidine [11,13]. Pyoverdines are fluorescent siderophores of pseudomonads that play important roles for growth under iron-limiting conditions, permitting their colonization of hosts, from humans to plants [13]. On the other hand, indigoidine is a blue diazadiphenoquinone pigment that can play an important role in tolerance to oxidative stress [14]. Recently, it was found that the pigmentation and the biofilm-forming ability of *P. fluorescens* are promoted at low incubation temperatures, suggesting their possible involvement in the spread and persistence of these strains in the dairy environment [15,16].

*L. monocytogenes* and *P. fluorescens* are able to form biofilms on different surfaces including polystyrene and stainless steel, which are materials commonly used in the food industry [17,18,19]. Dairy plants’ surfaces can be colonized by various microbial species in biofilms, also because of the increase in bacterial tolerance to common sanitizers. Biofilms found in nature are generally formed by two or more microbial species. In fact, multi-species biofilms are commonly encountered in food and food-related environments [20]. Multi-species interactions and the physiological conditions of microorganisms influence the properties of the formed biofilms. Compared to mono-species, mixed-species biofilms are known to provide advantages to microorganisms such as the increase in tolerance against stressful conditions and the capability to degrade organic compounds [21]. However, in multi-species biofilms, microbial cells can interact both positively and negatively [22]. In particular, on the basis of the different bacterial counts and biofilm biomass, the interactions between the two species are evaluated as synergistic (beneficial), neutralistic (no influence), or competitive (deleterious). In fact, when the biomass or the bacterial counts of a dual-species biofilm are greater than the sum of the two single-species biofilms under similar incubation conditions, the interaction is considered synergistic. On the other side, the interaction is evaluated as neutralistic if the biomass or the counts are equivalent, and competitive if they are less than the sum of the two [23].

Although studies on multi-species biofilms are of relevant importance and closer to natural conditions, most of the studies on biofilm formation analyze single-species biofilms [24,25,26]. In fact, only a few studies on bacterial biofilms are based on food model systems that mimic real environments [27,28,29]. Therefore, the objective of this study was to evaluate dual-species biofilms formed by *L. monocytogenes* and *P. fluorescens* in a system simulating real conditions encountered in dairy processing by using: (i) surfaces of polystyrene and stainless steel; (ii) *L. monocytogenes* and blue pigmenting *P. fluorescens* strains isolated from dairy products; (iii) Ricotta-based dairy model as the growth medium; and (iv) 12 °C as the incubation temperature.

## 2. Materials and Methods

### 2.1. Bacterial Strains

Eight strains of *Listeria monocytogenes* were tested together with one strain of *Pseudomonas fluorescens* (pf5), isolated from Mozzarella cheese and chosen for its capability to form biofilm and to produce blue pigment on Potato Dextrose Agar, Agar Mascarpone, and Mozzarella cheese [16]. *Listeria monocytogenes* strains previously isolated from dairy products (LM 1-2-3-4) and dairy plants (LM 5-6-7-8) were characterized and typed in this study. All of the strains were of Italian origin. 

The strains were maintained at −80 °C with an anti-freezing agent (glycerol, 20% *v/v*, Sigma) to preserve the viability of the cells during storage.

### 2.2. Characterization and Typing of L. monocytogenes Strains

The strains of *L. monocytogenes* were characterized and typed according to two commonly used techniques: serotyping and Pulsed-Field Gel Electrophoresis (PFGE) [30,31].

Serotyping was carried out according to US Food and Drug Administration (FDA) Bacteriological Analytical Manual [32] using commercial antisera for flagellar and somatic antigens (Denkan Seikem Co. Ltd., Tokyo, Japan), following the manufacturers’ instructions.

PFGE typing was carried out according to the Centers for Disease Control and Prevention PulseNet protocol (CDC, PulseNet Methods PNL04), employing the restriction enzymes ApaI and AscI (New England BioLabs Inc., Ipswich, MA, USA). *Salmonella enterica* Braenderup H9812 digested with the restriction enzyme XbaI (New England BioLabs) was used as the reference size standard. The separation of restricted DNA fragments was performed for 20–22 h in 1.5% (*w/v*) Agarose gels in 0.5× Tris-Borate-EDTA buffer (5× TBE diluted in MilliQ water) at 14 °C, 6 V cm^−1^ with switch times of 4–40 s through CHEF Mapper^®^ XA (Bio-Rad, Hercules, CA, USA). The images were acquired by using ChemiDoc^TM^ MP Imaging System (Bio-Rad, CA, USA) and the pulsotypes were analyzed with BioNumerics software version 7.5 (AppliedMaths, Sint-Latem, Belgium).

### 2.3. Inoculum

The strains were inoculated into Tryptic Soy Broth (TSB, Liofilchem, Roseto, Italy) from fresh microbial cultures, followed by overnight incubation at 37 °C (*L. monocytogenes*) and 30 °C (*P. fluorescens*). The bacterial cells were harvested by centrifugation (13,000 rpm for 5 min), washed three times with PBS (Phosphate Buffer Saline) solution, and the Optical Density (OD) of bacterial suspensions was measured by a spectrophotometer Lambda bio 20 (Perkin Elmer, Waltham, MA, USA) to obtain a cell count of about 10^5^ CFU/mL in the growth medium [33]. In order to maintain the same load for the mono- and multi-species inocula, individual bacterial suspensions were diluted in a 1:1 ratio with the medium for the mono-culture, and with the other microbial species for the multi-culture.

### 2.4. Ricotta-Based Medium Preparation 

The Ricotta-based dairy model was prepared following the method described by de Carvalho et al. [34] with some modifications. In detail, 160 g of Ricotta purchased from a local market were diluted in 1 L of distilled water, heated at 42 °C for 50 min in a water bath, and then autoclaved at 121 °C for 15 min. The broth was separated from the solid part by a sterile gauze and stored at 4 °C until use. 

### 2.5. Biofilm Formation on Polystyrene Microplates

To examine the biofilm-forming capability of the strains in mono- and dual-species culture and to select one combination, biofilm formation was assessed on polystyrene surface (PS microtitre plate, Corning incorporated, Kennebunk, ME, USA) for each strain. Then, the eight *L. monocytogenes* strains were combined with the blue pigmenting *P. fluorescens* strain. The bacterial suspensions, previously prepared in the Ricotta-based medium, were aliquoted (200 µL) into the wells of PS plates and then incubated at 12 °C for 168 h. Negative control wells contained non-inoculated Ricotta medium. Plates were incubated for 0, 48, 72, 96, and 168 h to allow biofilm formation, and after the incubation period planktonic cells were removed by aspiration and washing the wells with PBS. Thereafter, total biomass (cells plus matrix) was quantified at 590 nm by crystal violet assay, as reported by Rossi et al. [16].

Means and standard deviations of absorbance values derived from five replicates were calculated by subtracting the control value from each mean value. 

The blue pigment color appearance during the assay was evaluated visually as presence/absence.

### 2.6. Biofilm Formation on Stainless Steel and Enumeration of Planktonic and Sessile Cells

To evaluate biofilm formation on stainless steel, AISI 304 coupons (SS coupons, 2 × 2 × 0.1 cm), previously cleaned according to the procedure described by Campana et al. [35] and sterilized at 121 °C for 15 min, were used. *L. monocytogenes* LM5 strain was chosen as representative of the whole set and combined with *P. fluorescens* pf5. Each coupon was individually introduced into a sterile glass container, and then 5 mL of Ricotta medium were inoculated with the mono- or dual-species inocula. Also, negative control samples with non-inoculated Ricotta medium were included. The samples were then incubated at 12 °C for 168 h. The assay was performed in triplicate. At each sampling time (0, 48, 72, 96, and 168 h), three sterile glass containers were used. One milliliter of the suspension was taken from each sterile glass container, and serial dilutions were performed to enumerate planktonic cells. The dilutions were distributed on selective media for *Pseudomonas* spp. (Pseudomonas Agar Base; Oxoid-Thermofisher, Rodano, Italy) and for *L. monocytogenes* (Agar Listeria according to Ottaviani and Agosti; Biolife, Milano, Italy). The plates were incubated at 30 °C and 37 °C for 48 h, respectively for *P. fluorescens* and *L. monocytogenes*, and the number of colonies was counted. The enumeration of cells in biofilms (sessile cells) was performed by rinsing three times the SS coupons with saline solution (0.85% NaCl *w/v*) in sterile tubes, followed by scraping with two cotton swabs to collect the cells [36]. The swabs were immersed in 10 mL of saline solution, vortexing for 10 s at 230 rpm. Then, tenfold serial dilutions were prepared, and the colonies count was determined on the above mentioned agar media. 

The blue pigment color appearance during the assay was evaluated visually as presence/absence.

### 2.7. EPS Extraction from Biofilms and Total Carbohydrates Quantification

The determination of carbohydrates from biofilms formed on SS coupons was carried out in triplicate for the samples and the incubation times reported in Section 2.6.

The EPSs were extracted as previously reported by Abdallah et al. [37], with some modifications. Briefly, after rinsing, biofilm was scraped from the SS coupon by using cotton swabs that were immersed in 10 mL of saline solution (0.85% NaCl *w/v*). After that, the solution was sonicated for 5 min at 50 kHz (Starsonic 90 Digit, Liarre, Asti, Italy), the cells were removed by centrifugation (5000× *g* for 15 min), and the supernatant was collected. 

Carbohydrates quantification was carried out following the anthrone method [38] using 1 mL of the EPS extract. Then, 1 mL of the sample was transferred into a Pyrex test tube and 1 mL of cold anthrone reagent (0.1% anthrone solution in 75% sulfuric acid (*v/v*)) was added. The tubes were placed in a water bath (100 °C, 14 min) and were then cooled at 5 °C for 5 min. Glucose solution (100 mg/L) was used as a standard. The absorbance of the samples at 625 nm was measured, and the results were presented in µg/cm^2^. 

### 2.8. Confocal Laser Scanning Microscopy (CLSM) Analysis

Mono- and dual-species biofilm structures were observed by CLSM according to the method described by Rossi et al. [39]. Briefly, cultures of mono- and dual-species of *L. monocytogenes* LM5 and *P. fluorescens* were prepared in Ricotta-based medium as reported in Section 2.3 and were inoculated in eight-well chamber slides (Nunc, Thermo Fisher Scientific, Waltham, MA, USA) at 400 mL per well. A negative control with non-inoculated medium was prepared. The plates were incubated at 12 °C, and the biofilms were allowed to grow for 48, 72, 96, and 168 h without agitation. At each time point, the pre-formed biofilms in each well of the plate were rinsed with sterile water to remove the growth medium and the unattached cells. The biofilms were stained with LIVE/DEAD BacLight Bacterial Viability kit containing the dyes SYTO9 and propidium iodide (PI) according to the manufacturer’s instructions (Molecular Probes, Thermo Fisher Scientific, Eugene, OR, USA). Live bacterial cells appeared fluorescent green, while dead/damaged bacterial cells appeared fluorescent red. The stained biofilms were observed with the Nikon A1R confocal imaging system and controlled by the Nikon NIS Elements software ver. 4.40 (Nikon Corp., Tokyo, Japan), equipped with a Plan Apo 100× oil objective. 

The excitation/emission for the dyes were 488/525–50 nm and 561.5/595–50 nm for SYTO9 and PI, respectively. The fluorescence of pyoverdine, the siderophore produced by *P. fluorescens*, was checked with the excitation/emission at 405/460 nm [40]. 

### 2.9. Statistical Analysis

Statistical analysis was performed using XLSTAT ver. 2017 (Addinsoft, Paris, France). The data were subjected to analysis of variance (ANOVA), and a Dunnett’s test was employed to compare single- and multi-species results. Statistical significance was achieved at * *p* < 0.05. 

## 3. Results

### 3.1. Serotype and Pulsotype of L. monocytogenes Strains

Four serotypes (1/2a, 1/2b, 1/2c, and 4b) were identified among the eight *L. monocytogenes* strains (Table 1). The most prevalent serotype was 1/2b (for strains isolated from both food and environmental sources), then 1/2a (for food strains) and 1/2c (for environmental strains), followed by 4b (for Mozzarella cheese isolate). A total of eight ApaI and eight AscI PFGE types were distinguished, thus revealing that the strains isolated from food products and environment were genetically different and heterogeneous.

### 3.2. Biofilm Formation on Polystyrene Surface

**Figure 1:**
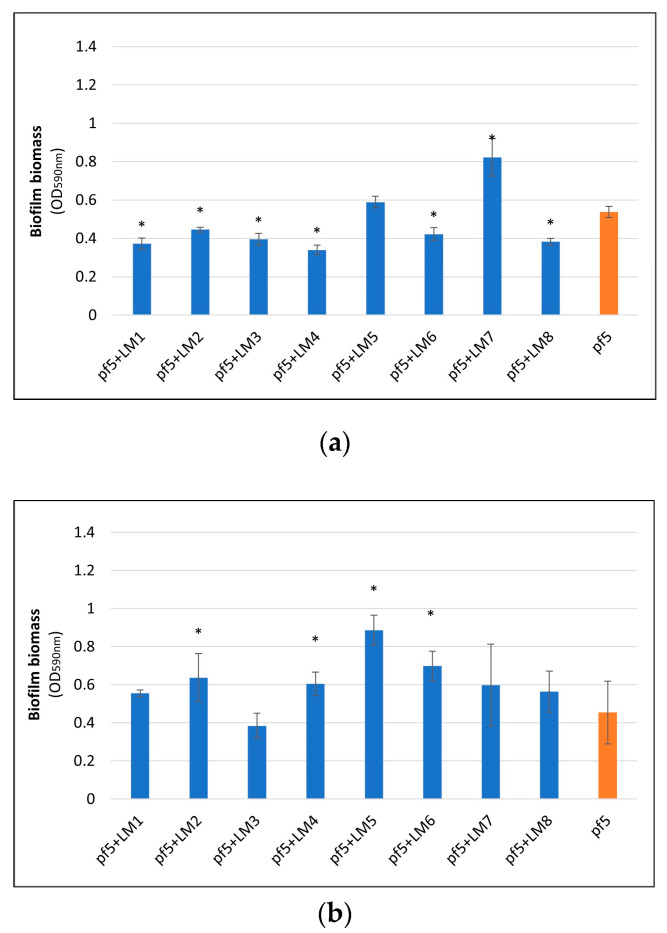

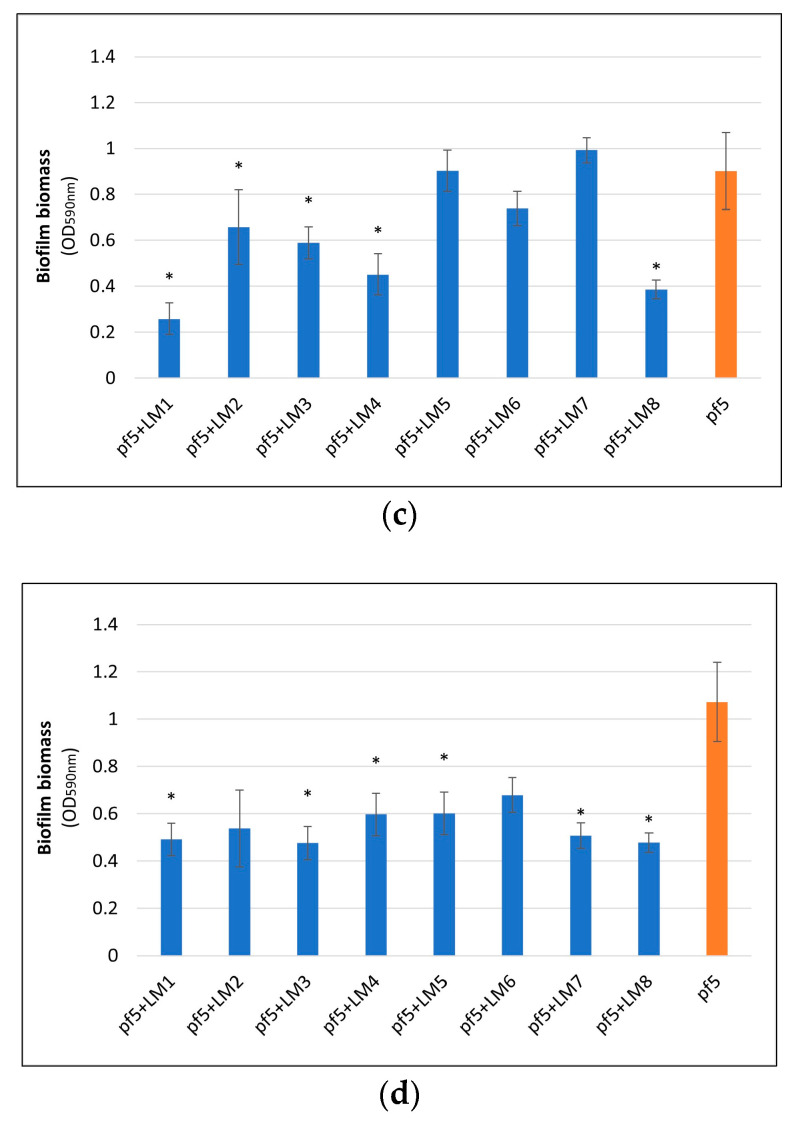
Biofilm biomass (OD_590 nm_) of *P. fluorescens* pf5 in mono- and dual-species with *L. monocytogenes* strains on a polystyrene (PS) surface at 12 °C for 168 h. The results are expressed as an average of five replicates and the bars represent the standard deviations. The asterisk (*) indicates statistically significant difference between the mono- and dual-species samples for the same incubation time (* *p* < 0.05). (**a**) 48 h, (**b**) 72 h, (**c**) 96 h, and (**d**) 168 h.

The results of biofilm formation ability of *P. fluorescens* pf5 in mono- and dual-species with *L. monocytogenes* strains on PS surface are shown in Figure 1. None of the eight *L. monocytogenes* strains was able to form biofilms on PS (data not shown). On the other hand, *P. fluorescens* exhibited good biofilm formation capacity. In fact, the biofilm biomass of *P. fluorescens* pf5 in single-species increased during incubation time, reaching a maximum value after 168 h of incubation (OD_590 nm_ 1.072 ± 0.167). However, a different behavior was observed for the species in combination, with biofilm biomass variability among the strains. Although at the end of the incubation period biofilms in dual-species systems were significantly lower than the single ones (* *p* < 0.05), a higher biofilm biomass for some strains was noticed after 72 h, particularly for the combinations *P. fluorescens–L. monocytogenes* LM5 (Figure 1b). With respect to the blue discoloration, *P. fluorescens* blue pigment production was monitored during the whole incubation time, and after 72 h the color change of the substrate was observed both in single- and mixed-culture (Supplementary Figure S1). 

Based on the obtained results, the combination *L. monocytogenes* LM5 and *P. fluorescens* pf5 was selected for the subsequent analysis.

### 3.3. Biofilm Formation on Stainless Steel Surface and Enumeration of Planktonic and Sessile Cells

**Figure 2:**
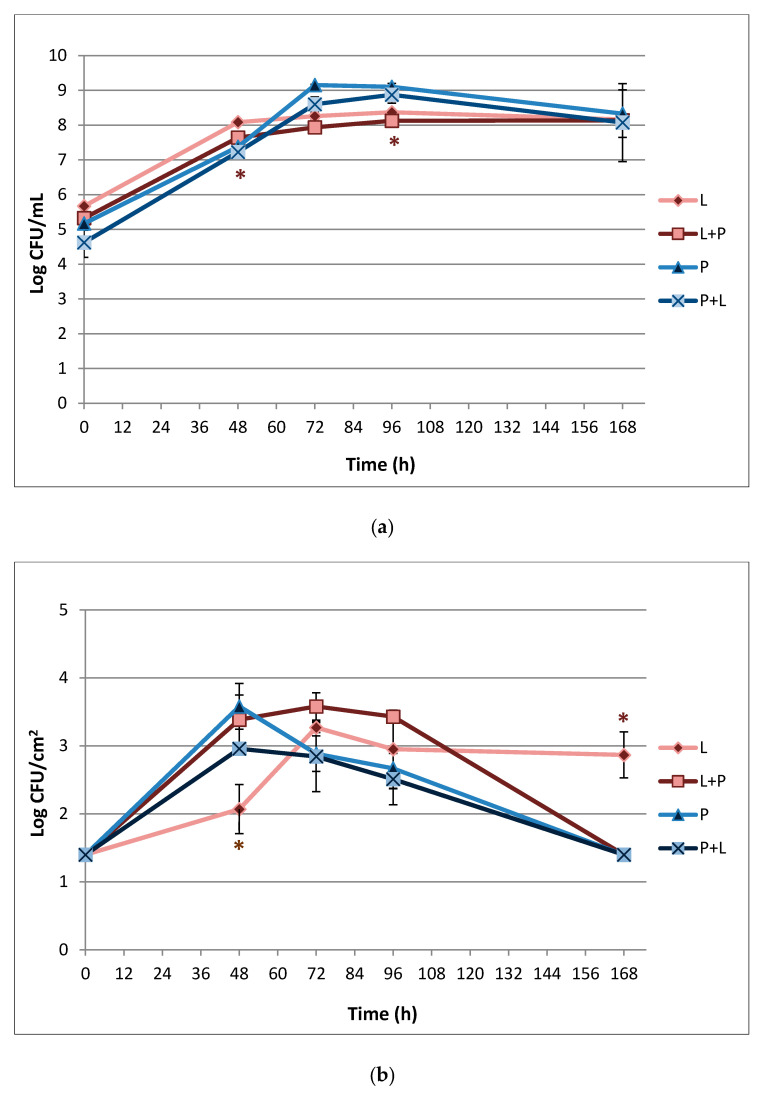
Dynamics of planktonic (**a**) and sessile (**b**) cells of *L. monocytogenes* LM5 and *P. fluorescens* pf5 in mono- and dual-species conditions on stainless steel (SS) coupons at 12 °C for 168 h. The results represent average values of three replicates and the bars indicates the standard deviations. The asterisk (*) means statistically significant difference between the mono- and dual-species of each strains for the same incubation time (* *p* < 0.05). L: *L. monocytogenes* in single-species; L + P: *L. monocytogenes* in dual-species; P: *P. fluorescens* in single-species; P + L: *P. fluorescens* in dual-species.

The results of *L. monocytogenes* LM5 and *P. fluorescens* pf5 planktonic and sessile cells on SS coupons in both mono-culture and dual-culture conditions, are presented in Figure 2.

Regarding the planktonic phenotype (Figure 2a), under mono-species, starting from a load of 5.67 ± 0.08 Log CFU/mL *L. monocytogenes* reached 8.16 ± 0.06 Log CFU/mL after 48 h, remaining almost stable until the end of the incubation time. However, the presence of *P. fluorescens* determined a slight but significant (* *p* < 0.05) decrease of *L. monocytogenes* counts of about 0.35 and 0.24 Log CFU/mL at 48 and 96 h, respectively. *P. fluorescens* showed a greater increase in load over time compared to *L. monocytogenes*: 5.17 ± 0.14 Log CFU/mL at time 0, 7.3 ± 0.06 after 48 h, and 9.15 ± 0.02 Log CFU/mL at 72 h, followed by a load reduction of about 1 Log CFU/mL. *P. fluorescens* planktonic counts did not differ significantly between mono- and dual-species conditions. 

The results regarding the sessile populations (Figure 2b) showed that *L. monocytogenes* was able to adhere on the SS surface. In fact, *L. monocytogenes* in mono-species starting from a load of about 1.4 Log CFU/cm^2^ at time 0 increased up to 3.27 ± 0.07 at 72 h, maintaining these values as almost stable over time. In multi-species conditions, the presence of *P*. *fluorescens* statistically (* *p* < 0.05) increased the pathogen biofilm population after 48 h of incubation, when it reached a sessile load of 3.39 ± 0.36 Log CFU/cm^2^. However, at the end of the experimental time, *L. monocytogenes* sessile cells in mixed-culture dropped to 1.4 Log CFU/cm^2^. 

For the sessile population, the culture conditions (mono- or dual-species) did not influence *P. fluorescens* population level. In fact, it reached a sessile load of 3.58 ± 0.34 Log CFU/cm^2^ at time 48 h, which decreased during the time reaching 1.4 Log CFU/cm^2^ after 168 h, with no statistically significant differences among single- and mixed-culture. 

**Figure 3:**
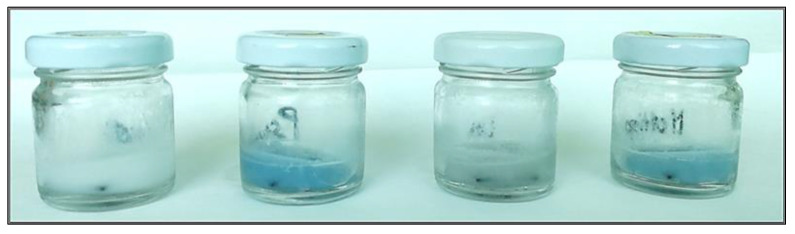
Blue pigment color appearance during the assay of biofilm formation on SS coupons using glass container (72 h, 12 °C). From the left: Control, *P. fluorescens* pf5, *L. monocytogenes* LM5, and dual-species system.

Also, in this experiment, the blue pigment production of *P. fluorescens* pf5 was observed both in single- and in mixed-culture starting from 72 h (Figure 3), when the highest load of the spoilage bacteria was detected (9.15 ± 0.02 and 8.60 ± 0.21 Log CFU/mL, respectively for pf5 in single- and multi-species conditions).

### 3.4. EPS Analysis by Carbohydrates Quantification

**Figure 4:**
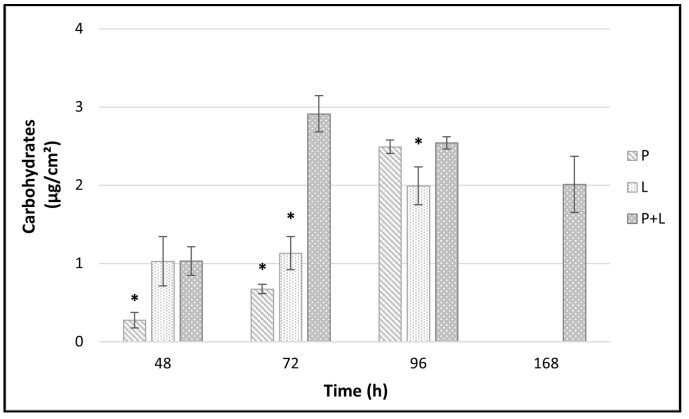
Carbohydrates amount (µg/cm^2^) from *L. monocytogenes* LM5 and *P. fluorescens* pf5 biofilms on SS coupons in mono- and dual-species at 12 °C for 168 h. The results indicate the average of three values and the bars indicate the standard deviation. The asterisk (*) means statistically significant difference between the mono- and dual-species of each strains for the same incubation time (* *p* < 0.05). P: *P. fluorescens* in single-species; L: *L. monocytogenes* in single-species; P + L: *L. monocytogenes* and *P. fluorescens* in dual-species.

Quantification (µg/cm^2^) of the carbohydrates extracted from the EPS of *P. fluorescens* pf5 and *L. monocytogenes* LM5 biofilms is shown in Figure 4. The total amount of carbohydrates in the biofilms was affected by the time and the species involved in biofilm formation. In single-species biofilms, the biofilm carbohydrates content increased over time with the greatest increase occurring between 48 and 96 h. In fact, at 96 h, values of about 2.49 ± 0.08 and 1.99 ± 0.24 µg/cm^2^ were observed for *P. fluorescens* and *L. monocytogenes*, respectively. Instead, no carbohydrates were revealed at 168 h for both single-species biofilms. The dual-species interactions in biofilms did not have any additive effect on carbohydrate matrix but a statistically significant (* *p* < 0.05) higher yield than those of the single-species was detected at time 72 h (2.91 ± 0.23 µg/cm^2^). Remarkably, the carbohydrates of the dual-species biofilms were revealed at high amount (about 2 µg/cm^2^) also at the end of the experiment (168 h).

### 3.5. Confocal Laser Scanning Microscopy Analysis

**Figure 5:**
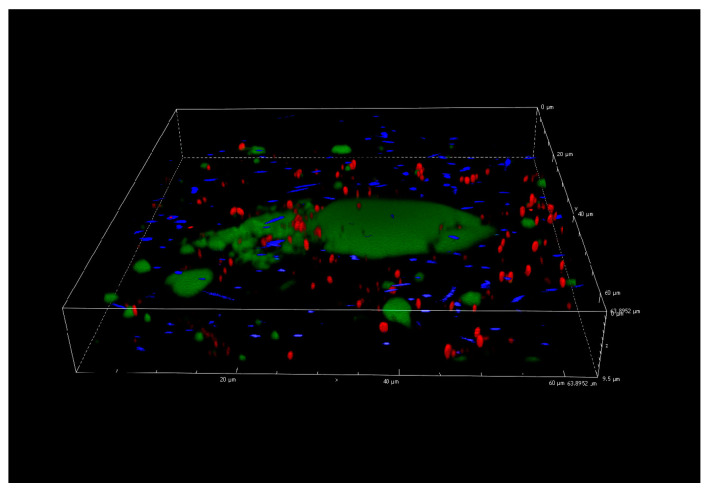
Confocal Laser Scanning Microscopy (CLSM) analysis of *L. monocytogenes* LM5 and *P. fluorescens* pf5 biofilms in dual-species conditions after 168 h at 12 °C.

**Figure 6:**
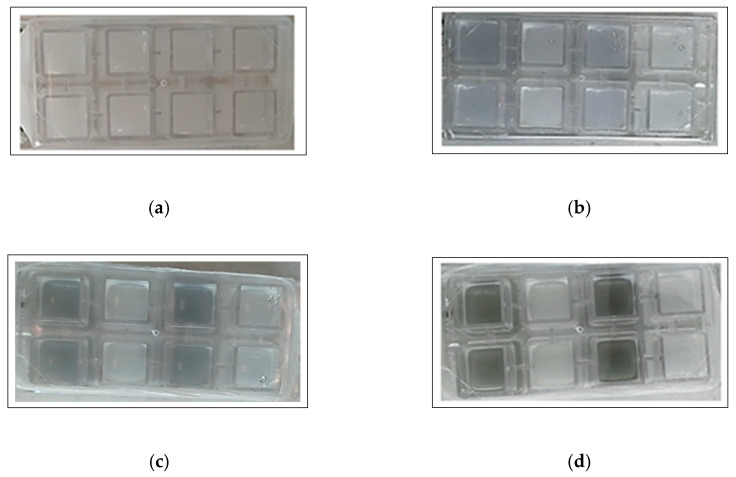
Blue pigment color evolution during the CLSM analysis using the Nunc wells: (**a**) 48 h, (**b**) 72 h, (**c**) 96 h, and (**d**) 168 h.

Figure 5 describes the dual-species biofilms at the end of the incubation period (168 h, 12 °C), on the basis of CLSM results. No three-dimensional biofilm architecture was revealed but, as for the other incubation times (data not reported), some unexpected evidences made it difficult to analyze the interactions between the two species on biofilm structure. In fact, the figure shows particular green agglomerates with damaged or dead cells (red cells according to PI staining) and detached *P. fluorescens* cells (blue color of pyoverdine fluorescence). The fact that the agglomerates were not clearly identifiable as cells and that they were present only in the samples with *P. fluorescens* pf5, suggests that they could depend on blue pigment appearance. In addition, this particular behavior was observed starting from 72 h in correspondence with the blue pigment formation by *P. fluorescens* (Figure 6). 

Figure 6a–d illustrates the color changes occurred in the CLSM plates during the incubation. Also, in this case, the blue pigment discoloration was revealed starting from 72 h, after which the blue color turned progressively into green/grey, particularly at 168 h.

## 4. Discussion

The biofilm forming capability of *P. fluorescens* and *L. monocytogenes* strains was evaluated in conditions simulating those encountered in a dairy environment. The serotypes of *L. monocytogenes* strains agree with previous observations, which showed that 1/2a, 1/2b, 1/2c, and 4b are the most common serotypes in foods and environmental sources [30], with 1/2a, 1/2b, and 4b being mainly responsible for listeriosis outbreaks [41]. 

The results obtained on the PS surface revealed that the *L. monocytogenes* strains used in this study were not able to form biofilms. Although numerous studies have demonstrated that this pathogen is able to form biofilms on various abiotic and biotic surfaces [42,43,44], also a previous study on mono-species and mixed-species biofilms composed of these two microbial species, reported very low OD values for mono-species *L. monocytogenes* biofilms, with a maximum value of 0.11 after 96 h of incubation at 20 °C [45]. On the contrary, *P. fluorescens* pf5 exhibited good biofilm formation capacity, which is an important feature for a spoilage organism. Cells in biofilms are an important risk factor in food environments for the potential contamination of foods and the possibility to become resistant to antimicrobials [20]. *Pseudomonas* spp., especially *P. fluorescens*, is a specific spoilage microorganism of dairy products and is frequently isolated from dairy environments [2,46,47]. 

In general, the population densities of the biofilms on the SS surface observed in our study were lower than those found by other studies [48,49,50], probably due to the conditions adopted to mimic dairy processing environments. In fact, the physical–chemical properties of substrates can affect surface bacterial adhesion and biofilm formation as highlighted by Parkar et al. [51], who observed an attachment decrease of both vegetative cells and spores of thermophilic bacteria on SS surfaces coated with milk proteins. 

The fact that *L. monocytogenes* LM5 was able to adhere to SS and not to PS could be attributable to the affinity established between the charge of the cell surface and the anchoring site. In fact, at low temperatures, *L. monocytogenes* increases the cell wall hydrophilicity and therefore the affinity to hydrophilic surfaces such as steel or glass [52]. 

In addition, the results revealed that dual biofilms were colonized to a greater extent by *L. monocytogenes* LM5 and that dual-species conditions lead to an increase in *L. monocytogenes* LM5 load compared to mono-species after 2 days of incubation. Stimulation of *L. monocytogenes* adhesion in mixed-culture biofilms with *P. fluorescens* was also described by Puga et al. [45], who linked the positive effects on *L. monocytogenes* to *Pseudomonas* production of proteinases, able to mobilize essential amino acids. Interestingly, Teh et al. [53] demonstrated that the proteolytic and lipolytic activities of bacterial cells in dairy environments are higher within biofilms than in planktonic cells. Moreover, the exopolymeric substances produced by *P. fluorescens* may facilitate *L. monocytogenes* anchorage, attachment, and surface colonization [54,55], providing a thick matrix protection [45]. Our findings are also consistent with the results of Hassan et al. [56], who observed a stronger attachment of *L. monocytogenes* to surfaces with preexisting *Pseudomonas* biofilms than to *Pseudomonas*-free surfaces. In contrast, in another study, the cell density of *L. monocytogenes* in dual-species biofilms formed at 15 °C for 48 h was lower than that of the single-species [48]. 

In our study, the interactions between the two microbial species led to a polysaccharides matrix over-production that persisted until the end of the experimental time (168 h). With this respect, Puga et al. [45] also reported that the inclusion of *L. monocytogenes* in the already established *P. fluorescens* biofilm increased matrix production. Although *L. monocytogenes* is considered “a cheater” with a poor matrix production capability, finding shelter inside the matrix of *Pseudomonas* spp., we observed good amounts of carbohydrates also in single-species *L. monocytogenes* biofilm. 

The fast cellular dispersal observed in our study for multi-species biofilms could have been stimulated from the early achievement of high biofilm level (cellular density/matrix threshold) with no extra nutrient supplementation [19,45,50]. As highlighted by the results of *L. monocytogenes* sessile cells on an SS surface (Figure 2b, Figure 4), the greater detachment rate of the cells in dual-species biofilms compared to the single ones was not associated with a poor EPS production. While Combrouse et al. [57] also did not observe any clear relationship between EPS content and CFU count, in contrast previous works reported a positive correlation between the ability to form biofilms and EPS production [58,59]. EPSs have a key role in maintaining the stability and integrity of biofilm structure via cellular adhesion and cohesion [60] but also other variables related to starvation and nutrient flow cessation [61] influence biofilms detachment.

With respect to the blue pigment production, as previously observed for Mozzarella cheese inoculated with *P. fluorescens* [16], Ricotta medium blue discoloration was observed at a high load of *P. fluorescens* pf5 after 72 h of incubation. In agreement with our findings, Andreani et al. [14] observed an evident blue pigment in broth when *Pseudomonas* counts reached about 7 × 10^8^ CFU/mL, concluding that the blue pigment production took place in the late logarithmic phase. Quintieri et al. [15] reported that in *Pseudomonas* spp. pigments modulate the transition from planktonic to biofilm state, show antimicrobial effects against other microorganisms, protect cells from oxidative stress, and can act as signaling molecules and virulence factors. Furthermore, Cude et al. [62], in a study conducted on marine *Roseobacter Phaeobacter* cells, found that indigoidine biosynthesis may provide a significant advantage in the colonization of environmental niches. In addition, the authors suggested that when a surface niche is colonized and quorum is reached, indigoidine biosynthesis is up-regulated to suppress the colonization of competing organisms. However, from our study it is not possible to state that the blue pigment produced by *P. fluorescens* pf5 possesses antimicrobial activity. In fact, during the blue pigment discoloration in mixed-culture, a strong reduction of *L. monocytogenes* load was observed only for the sessile cells at 168 h.

The color change from blue to green/grey highlights the possible reduction of indigoidine to leucoindigoidine [16], which is considered a chemical marker of blue discoloration [12]. Although the CLSM analysis did not allow for the detection of the architecture of single- and dual-species biofilms during the incubation time, the agglomerates formation and their interference with samples staining revealed some connection with *P. fluorescens* indigoidine production. In fact, the indigoidine and leucoindigoidine fluorescence was recorded using a 415 nm excitation and 520 nm emission wavelengths [63], which is near to the range of SYTO 9 used to observe live cells.

## 5. Conclusions

This study underlines the influence of microbial interactions on biofilms formed under simulated dairy processing conditions. The results showed that *L. monocytogenes* exhibited the capability to form biofilm only on SS coupons, while *P. fluorescens* formed biofilm on both PS and SS surfaces. The behavior of planktonic and sessile populations on SS coupons was dependent on the culture conditions. Particularly, the presence of *P. fluorescens* increased *L. monocytogenes* sessile population and total EPS carbohydrates amount on SS coupons. Green agglomerates, probably linked to the *P. fluorescens* blue pigment, were noticed during CLSM analysis and probably interfered with biofilm visualization. Nevertheless, further research is needed to better clarify this point and more studies would be useful to provide more information on the inter-species consortium. In fact, dual-species biofilms are more common than single-species ones in real conditions, show characteristics that could favor *L. monocytogenes* persistence in food production environments, and for this reason deserve thorough investigation.

## Figures and Tables

**Table 1:** Listeria monocytogenes and Pseudomonas fluorescens strains used in the study.

Species	Strain Name	Source of Isolation	Serotype	Pulsotype ApaI	Pulsotype AscI
*L. monocytogenes*	LM1	Gorgonzola cheese	1/2b	GX6A12.0051	GX6A16.0071
*L. monocytogenes*	LM2	Mozzarella cheese	4b	GX6A12.0073	GX6A16.0010
*L. monocytogenes*	LM3	Gorgonzola cheese	1/2a	GX6A12.0032	GX6A16.0029
*L. monocytogenes*	LM4	Caciotta cheese	1/2a	GX6A12.0390	GX6A16.0271
*L. monocytogenes*	LM5	Environmental	1/2b	GX6A12.0349	GX6A16.0255
*L. monocytogenes*	LM6	Environmental	1/2b	GX6A12.0005	GX6A16.0009
*L. monocytogenes*	LM7	Environmental	1/2c	GX6A12.0373	GX6A16.0261
*L. monocytogenes*	LM8	Environmental	1/2c	GX6A12.0002	GX6A16.0007
*P. fluorescens*	pf5	Mozzarella cheese			

## Data Availability

Data available on request.

## Supplementary Materials

The following are available online at https://www.mdpi.com/2304-8158/10/1/176/s1, Figure S1: Blue pigment production appearance during the assay of biofilm formation of *L. monocytogenes* strains and *P. fluorescens* pf5 in mono- and dual-species conditions on PS microtitre plates. (a) 0 h; (b) 24 h; (c) 48 h; (d) 72 h; (e) 96 h; (f) 168 h.
