# Supplementary material for: Interactions between L. monocytogenes and P. fluorescens in Dual-Species Biofilms under Simulated Dairy Processing Conditions
Source: Foods. 2021 Jan 16;10(1):176. doi: 10.3390/foods10010176 (PMC7829993; doi:10.3390/foods10010176)
Supplement: Supplementary file 1 [file foods-10-00176-s001.pdf]

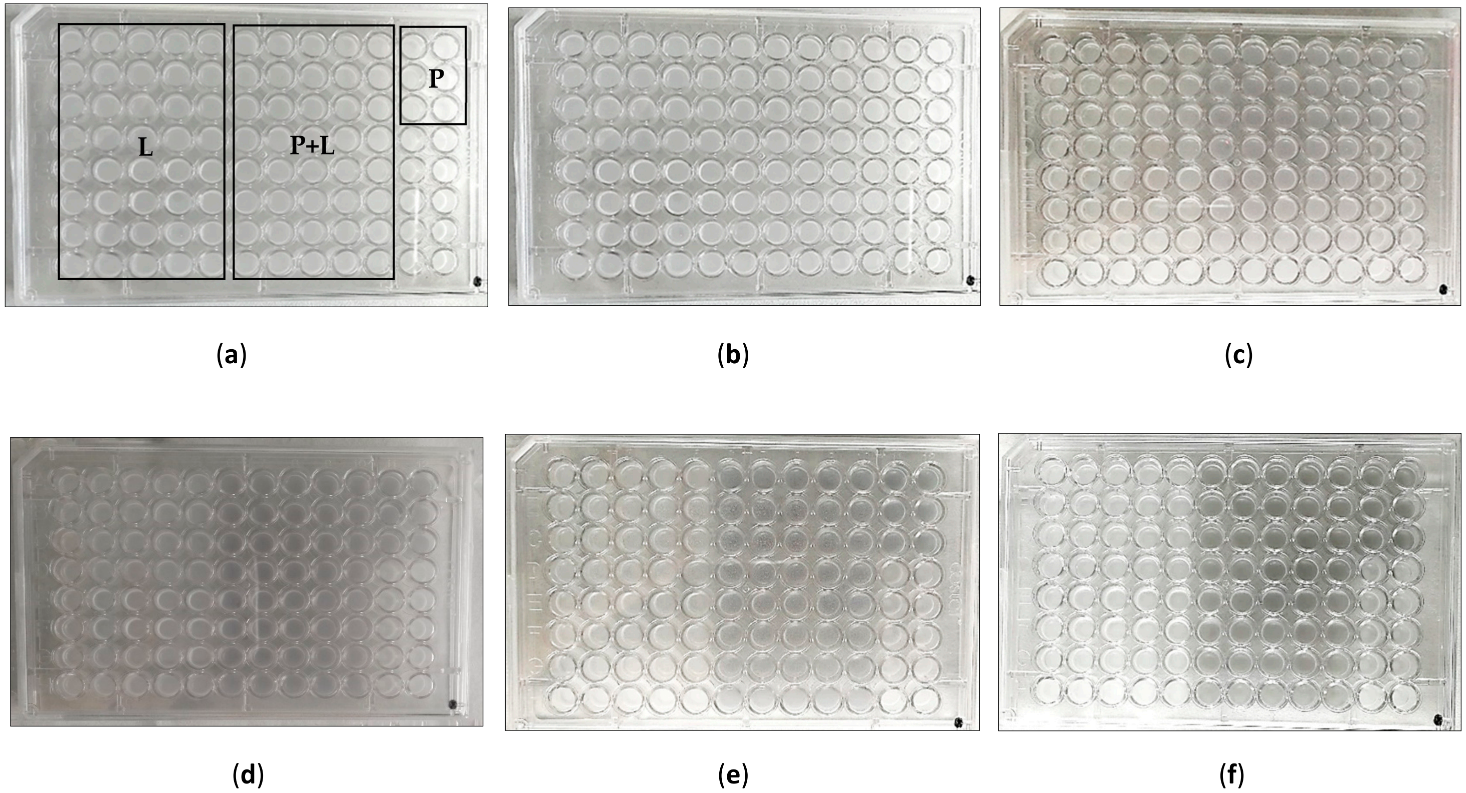

Figure 1S. Blue pigment production appearance during the assay of biofilm formation of *L. monocytogenes* strains and *P. fluorescens* pf5 in mono- and dual-species conditions on PS microtitre plates. (**a**) 0 h; (**b**) 24 h; (**c**) 48 h; (**d)** 72 h; (**e**) 96 h; (**f**) 168 h.
